# Long Term Cosmetic Application Improves Tactile Discrimination in the Elderly; a New Psychophysical Approach

**DOI:** 10.3389/fnagi.2019.00164

**Published:** 2019-06-28

**Authors:** Jean-Marc Aimonetti, Claire Deshayes, Marcel Crest, Pierre-Henri Cornuault, Benjamin Weiland, Edith Ribot-Ciscar

**Affiliations:** ^1^Aix Marseille University, CNRS, Laboratoire de Neurosciences Sensorielles et Cognitives (LNSC), SFR3C, Marseille, France; ^2^Aix Marseille University, CNRS, LNC, SFR3C, Marseille, France; ^3^Femto-ST Institute, Department of Applied Mechanics, University Bourgogne-Franche-Comte, CNRS/ENSMM/UFC/UTBM, Besançon, France

**Keywords:** tactile discrimination, haptic touch, skin aging, psychophysics, cosmetic

## Abstract

**Introduction**: Tactile sensitivity is impaired in older adults, which contributes to the loss of manual dexterity and mobility function. The reliability of classical psychophysical tests, such as two-point gap discrimination, has been questioned. Here we tested a new method to determine tactile acuity during dynamic touch, which is more functional than static touch. The aim was to validate a method providing a high level of discrimination of tactile acuity in the elderly.

**Methods**: We tested the ability of subjects to evaluate the distance between bands printed on poly-methyl-methacrylate (PMMA) sheets. Pairs of sheets were compared in two groups of participants aged from 60 to 74 years; the test group was required to apply a cosmetic foam with an active ingredient on both their hands twice a day for 1 month, the control group had an identical task but used the same cosmetic foam without any active ingredient. The tests were run in a double-blind, placebo-controlled study.

**Results**: The tactile discrimination threshold decreased by 83 μm after 1 month of cosmetic application in the group using the active ingredient, while it was unchanged in the control group.

**Discussion**: The test presented here provided highly accurate results and should be useful to determine tactile performance. It allows the monitoring of tactile rehabilitation and/or skin treatments used to restore tactile acuity in the elderly.

## Introduction

Tactile sensitivity relies on the sensory information provided by low-threshold mechanoreceptors located in the dermis of the glabrous skin, such as Merkel’s disks, Meissner, Pacinian, and Ruffini’s corpuscles. These mechanoreceptors are preferentially activated by deformation of the skin such as pressure, vibration, and tension. These mechanical events are transduced into sensory messages going through the peripheral and then the central nervous system, where their processing give rise to tactile sensations (Johnson, 2001).

Tactile sensitivity is known to decline with age, resulting from mechanoreceptor loss (Bruce, 1980; Iwasaki et al., 2003; Skedung et al., 2018), but also from changes in the mechanical properties of the skin itself such as reduced elasticity (Farage et al., 2013; Skedung et al., 2018), that may result from a decrease in hydration (Verrillo et al., 1998; Skedung et al., 2018). Thus, both tactile detection threshold and tactile acuity are impaired with age, where the feet and hands are the most affected, as a result of a reduced blood flow and/or greater physical wear on the contact surfaces (Thornbury and Mistretta, 1981; Stevens and Choo, 1996; Bowden and McNulty, 2013; da Silva et al., 2014; Franco et al., 2015). This has important functional implications for older adults, as it has been linked to impairment in manual control (Wickremaratchi and Llewelyn, 2006), but also deficits in balance and walking ability with an increased risk of injurious falls, which contributes to disability and death in older adults (Soriano et al., 2007). More tragically, many older adults are unaware of their peripheral neurological impairment and are therefore unlikely to seek preemptive intervention (Cruz-Almeida et al., 2014). In a previous study conducted in aged people, we demonstrated that tactile acuity increased 30 min after the application of a moisturizing cream (Lévêque et al., 2000). This short-term beneficial effect of skin hydration on tactile sensitivity has been confirmed in older men (Bowden and McNulty, 2013). These results suggest that, contrary to what has been claimed previously (Woodward, 1993), changes in the mechanical properties of the skin may affect tactile perception.

Tactile sensitivity is classically characterized by analyzing the detection threshold, with calibrated monofilaments, or by spatial discrimination, such as the two-point gap discrimination test (Bell-Krotoski et al., 1993). However, these tests have been criticized due to great variations within subjects, between subjects, and between studies (Levin et al., 1978; Lundborg and Rosén, 2004). For instance, calibrated monofilaments are simple to use, but they are fragile and can be distorted after multiple uses, as well as being sensitive to temperature and humidity (Haloua et al., 2011). This probably accounts for the unreliability of the tests notably those performed with the five smallest monofilaments, i.e., 0.008–0.16 g (Massy-Westropp, 2002). The results obtained with the two-point gap discrimination test are also extremely variable because they depend upon the way the test is performed, notably the amount of pressure applied to the skin and the synchronicity of application of the two stimulation points (Johnson and Phillips, 1981; Lundborg and Rosén, 2004). Recently, a test where the participant has to discriminate the orientation (horizontal vs. vertical) of two points of contact has been recommended as a better measure of tactile spatial acuity (Tong et al., 2013).

In the present study, we chose an active touch test, which is more functional than a static test, and evaluated a psychophysical method to assess spatial discrimination threshold. We designed a double-blind, placebo-controlled study in healthy elderly subjects to determine whether any beneficial effect of a cosmetic substance may be found, as compared to a placebo substance, by means of this psychophysical method. The demonstration of a difference between the two groups of participants in tactile acuity should validate the present method as a useful tool to track the beneficial effects of sensory training and/or treatment in older people.

## Materials and Methods

### Participants

The experiments were performed in 42 healthy, right-handed volunteers aged from 60 to 74 years. They were recruited in the retirement communities in the Marseille area of France. The exclusion criteria were: a history of neurological, psychiatric, or dermatological disorders, or clinically significant peripheral neuropathy, such as diabetes. No ethical review process was required for the present study because the French Conseil d’Etat ruled in an order dated 8 February 2017 that studies using cosmetic products, even for scientific investigation, do not require an application for approval from an ethics committee. Our study nevertheless conformed with the ethical guidelines set out by the Declaration of Helsinki and all participants gave their written informed consent.

### Design and Procedure

Participants were tested in a quiet room, with a constant temperature of ~22°C. They were asked to attend an initial test to evaluate their tactile acuity. The participants were seated comfortably in an armchair with their right hand positioned in a cushioned groove, which allowed a standardized and relaxed position. The participants were asked to close their eyes and they wore noise-canceling headphones (Bose; Framingham, MA, USA).

**Figure 1:**
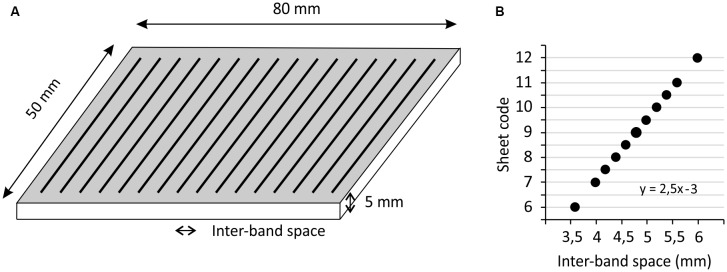
The subjects were required to explore pairs of poly-methyl-methacrylate (PMMA) sheets printed with regular grooves **(A)**, the inter-band space was constant in each sheet and varied around a reference sheet (large point in **B**).

Participants were asked to explore pairs of poly-methyl-methacrylate (PMMA) sheets specifically designed for the experiment, using the distal phalanx of their right index finger. These sheets, of 5 mm thickness and 50 × 80 mm^2^ surface, included regularly-spaced grooves with a depth of ~0.07 mm, which were oriented perpendicularly to the finger displacement (Figure 1A). The space between the grooves (the “inter-band space”) varied from 3.6 to 6 mm between test sheets. The sheet with a median spacing of 4.8 mm was defined as the reference. On each side of this reference, four test sheets with spacing varying in 0.2 mm steps were used. At each extreme, the sheets presented a change in the spacing of 0.4 mm. For convenience, the sheets were coded as follows: the shortest spacing (3.6 mm) was coded as 6; then the sheets with spacings of 4, 4.2, 4.4, 4.6 mm were coded as 7, 7.5, 8, 8.5, respectively; the reference (spacing of 4.8 mm) was coded as 9; then the sheets with spacings of 5, 5.2, 5.4, 5.6 mm were coded as 9.5, 10, 10.5, 11, respectively; finally the largest spacing (6 mm) was coded as 12 (Figure 1B). The method consisted of presenting pairs of sheets, where the reference was always presented together with one of the 10 test sheets, and the order of presentation was randomized for each pair. The participants were instructed to explore each sheet once, from top to bottom, at about 20 mm/s, which corresponds to the speed classically chosen to explore textured surfaces (Vega-Bermudez et al., 1991). They were familiarized with this instruction before the test session by moving their finger over the sheet during two audible beeps that were spaced 4 s apart.

The test was a two-alternative forced choice discrimination task, where the participants had to determine whether the first or the second sheet had the larger inter-band spacing. The eight sheets with a 0.2 mm-spacing difference were presented 15 times and the two extreme sheets with a 0.4 mm-spacing difference six times; the lower number of presentations for the two extreme sheets was justified by the ease in differentiating these sheets from the reference. In total, the participants had to compare 132 reference/test pairs. The whole psychophysical examination lasted about 1.5 h, including rest time of 5 min every 15 min.

After this initial tactile test (called “pre”), the participants received cosmetic foam (Laboratoires Chemineau, Anjac Health and Beauty Inc.). The participants were asked to apply the foam on their both hands (palm, fingers, and dorsum) each morning in their daily routine and each evening before sleeping, for 1 month. They were instructed not to wash their hands in the hour following the foam application.

The 42 participants were divided into two groups: “active” and “placebo.” The repartition was done in a random manner, taking into account the participant’s age and gender. Participants of the active group (*n* = 21, six men) received the foam with an active ingredient (IP patent in process n° BFF170321IMA, Laboratoires Chemineau, Anjac Health and Beauty Inc.). In the placebo group, participants received a foam with exactly the same formulation, apart from the active ingredient (*n* = 21, six men). The participants and the experimenter (CD) were blinded to who received the active formulation as both treatments were placed in similar packages only identified as A and B; the participants were even blinded as to the existence of any placebo, in order to enhance commitment with the experiment (Bang, 2016). The participants were then asked to attend a second time, exactly 30 days after application of the foam, to evaluate their tactile sensitivity during the “post” test that was run in exactly the same conditions as the pre-test.

### Data Analysis

In order to evaluate and compare participants’ performances across the two tests (Pre/Post), the psychometric data (i.e., the proportion of correct answers, corresponding to which sheet was of larger inter-band space) were fitted by the following cumulative Gaussian function:

$$P(x)=\lambda +(1-2\lambda )\frac{1}{\sigma_{\Psi }\sqrt{2\pi }}\int_{-\infty }^{x}e^{-\frac{{\left(y-\mu \Psi \right)}^{2}}{2\sigma_{\Psi }^{2}}}dy$$

**Figure 2:**
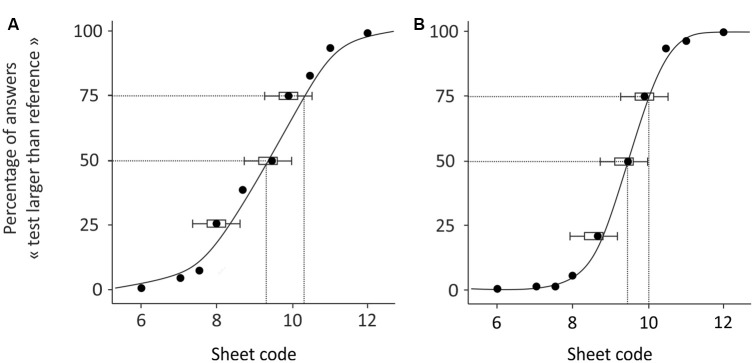
Examples of psychometric curves obtained from two subjects during the pre-test. The participant in **(B)** performed better than the participant in **(A)**.

Here, *x* represents the sheet code; μψ is the mean of the Gaussian, i.e., the point of subjective equality (PSE), that corresponds to the stimulation intensity (here, the inter-band space) leading the participant to perceive no difference between the reference and the test sheet; and σψ is the standard deviation of the curve (discrimination threshold), which is inversely related to the participant’s discrimination sensitivity. The σψ value (also called the uncertainty range) is given by the difference between the projection onto the X-axis of 75% and 50% of response (see Figure 2). A smaller σψ value corresponds to higher discrimination sensitivity in the task. The PSE is given by the projection at 50% of the response. The two indices, PSE and σψ, characterize the participant’s performance, and λ accounts for stimulus-independent errors (e.g., due to participant lapses) and was restricted to small values (0 < λ < 0.06, Wichmann and Hill, 2001). This parameter is not informative about the perceptual decision, thus we disregarded it for the subsequent analyses. Psignifit toolbox, implemented in MATLAB software (The Mathworks, Natick, MA, USA) was used to fit the psychometric curves.

Differences in the PSE and uncertainty range between the groups, before cosmetic treatment, were evaluated with an unpaired Student’s *t*-test. The impact of cosmetic application upon tactile sensitivity was estimated within each group with a paired Student’s *t*-test. Effect sizes were calculated according to Cohen’s instructions where *d* < 0.2 corresponds to a small effect, 0.2 < *d* < 0.5 a medium effect, and *d* > 0.5 a strong effect (Cohen, 1992).

## Results

### Basic Demographics and Clinical Characteristics

Among the 42 participants, a total of eight participants (four in each group) were excluded because their responses in the pre- and/or post-tests were almost indistinguishable from chance level. These results were explained either by an inability to perform the test both in pre- and post-tests (four subjects) or for medical reasons that interfered with the post-test after the 30 days of foam application (four subjects, lesion of the index, sickness, temperature etc.). Details of the basic demographics and clinical characteristics of the 34 participants finally included in the study are given in Table 1. As can be seen, the two groups of participants were similar, with regard to age and gender because the groups were established on the basis of these parameters, but also with regard to their socio-professional characteristics and medical treatments (no significant difference).

**Table 1:** Basic and clinical characteristics of the two groups of subjects tested (*n* = number).

	Active	Placebo	Mann-Whitney test
			*Z* limit = 1.96
**Basic demographics**			
Age (years)	69.0 ± 2.9	66.4 ± 3.9	*Z* = 1.60, *p* = 0.11
Men (*n*)	6	5	*Z* = 0.29, *p* = 0.77
Right-handers (*n*)	17	17	*Z* = 0, *p* = 1
Senior executive, teacher (*n*)	3	5
Technician (*n*)	7	4	Not applicable
Workers (*n*)	7	8
**Clinical characteristics**			
No treatment (*n*)	8	9	*p* = 1.06
Hypertension (*n*)	5	5
Cholesterol (*n*)	3	6
Thyropenia (*n*)	3	1	
Arrythmia (*n*)	2	1
Other drug therapies (*n*)	5	4

### Psychophysical Results

Figure 2 illustrates the results obtained in two participants during the pre-test. The results reported in Figure 2A shows that the subject answered correctly for the sheets coded 6–7.5, where none of these had an inter-band space larger than that of the reference, and the percentage of responses deemed “larger” was close to 0%. The responses are less certain for sheets 8–10, where the inter-band space was close to that of the reference and the percentage of response is around 50%. The performance improved with sheets 10.5–12, where, for most of the time, the participant answered correctly that the test sheet had larger inter-band spacing than the reference, and the percentages of responses reached 100%. From this psychometric curve, we extracted the PSE, which here equaled 9.1, and the uncertainty range was 1.25. Therefore, for this participant, the minimal inter-band space for a sheet to be statistically differentiated from the reference was at least 0.5 mm. The results reported in Figure 2B are those of a participant who performed the task better, as shown by the psychometric curve, which had a steeper slope than that in Figure 2A. More precisely, their responses were uncertain only for sheets 9.5 and 10. The PSE remained close to the reference (9.4) and the uncertainty range was smaller (0.81), meaning that only 0.32 mm of difference between two sheets was necessary for these to be differentiated by this participant, who thus presented a better tactile discrimination.

At the population level, the PSE was not found to differ during the pre-test between the active and placebo groups (9.3 ± 0.4, 9.2 ± 0.3, respectively, *t* = 0.85, *p* = 0.4). After 1 month of cosmetic application, the PSE calculated in each group during the post-test was not found to differ with that calculated during the pre-test, either in the active group (9.2 ± 0.3, *t* = 0.98, *p* = 0.34) or in the placebo group (9.4 ± 0.5, *t* = 1.67, *p* = 0.11). In other words, in both groups, applying cosmetic foam for 1 month did not affect the tactile evaluation of the reference sheet.

**Figure 3:**
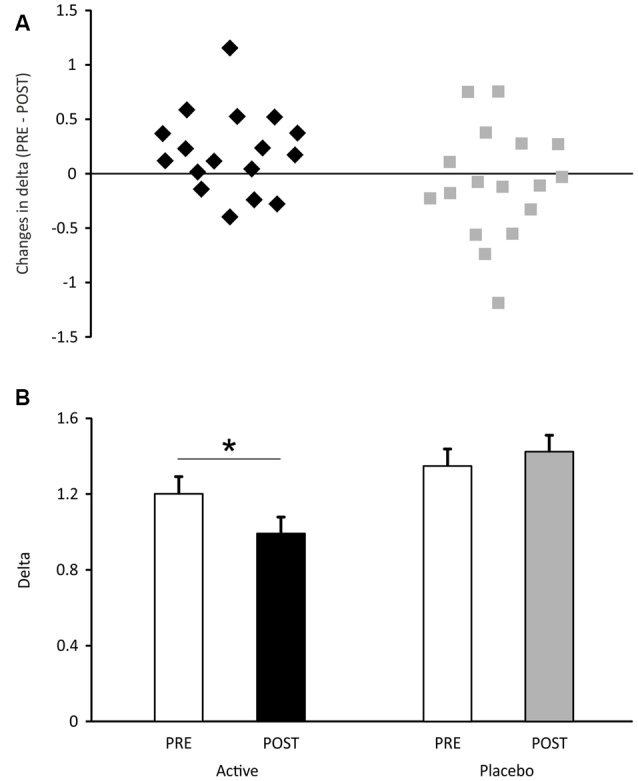
Uncertainty range measured in the active and placebo groups of participants. In **(A)**, the mean data are presented (±SEM). In **(B)**, the individual subject data are expressed as the difference between uncertainty ranges measured during pre- and post-tests. Positive values express an increase in tactile discrimination. *p < 0.05.

With regard to the uncertainty range (Figure 3), it was not found to differ between the two groups in the pre-test (*t* = 1.14, *p* = 0.26). However, in the post-test, the uncertainty range was significantly smaller than that measured during the pre-test, in the active group (Figure 3A, *t* = 2.41, *p* = 0.03, *d* = 0.57) and remained unchanged in the placebo group (*p* = 0.54). This means that, in the active group, a minimal difference of 0.48 mm in the inter-band space, that was necessary for a significant discrimination, fell to 0.40 mm after cosmetic application. In other words, a decrease of 83 μm in spatial discrimination followed 1 month of cosmetic application.

## Discussion

The present study aimed at evaluating the reliability of a new psychophysical method to assess tactile acuity in the elderly. We compared the impact of 1-month application twice a day of a cosmetic substance, which did or did not include an active ingredient favoring skin hydration. The spatial discrimination threshold was assessed in a double-blind, placebo-controlled manner, where we tested the ability of participants at discriminating a test surface with grooves either more distant or closer than those of a reference surface, during active touch. The results showed that the spatial discrimination acuity was significantly improved in the group of participants treated with the active ingredient, while it was unchanged in the placebo group. Importantly, the two groups of participants were very similar in terms of their spatial tactile acuity in the pre-test, eliminating any group differences on the observed improvement. This must be noted, since people are not equal regarding skin aging and their related decline in tactile acuity, as some elderly remains as high tactile performers, comparable to younger people (Skedung et al., 2018). In the present population of participants, there was a variability in tactile abilities and therefore were also included the high performers, but their presence was counterbalanced in both groups.

Tactile sensitivity is classically characterized by analyzing the detection threshold, with calibrated monofilaments, or the spatial discrimination, using the two-point gap discrimination test. It has been reported that, after moisturizing the skin in seniors, the former decreases by 50 mg and the latter by about 3 mm (Lévêque et al., 2000; Bowden and McNulty, 2013). In both studies, the effects of skin hydration were evaluated only 30 min after cosmetic application. Besides methodological issues, such as the unreliability of force with the smallest monofilaments or the synchronization of application with the two-point gap discrimination test (Levin et al., 1978; Lundborg and Rosén, 2004), these tests evaluate only static touch and not dynamic touch. Presently, we focused on the more functional characteristics (i.e., dynamic touch), because during active touch, proprioceptive inputs implement cutaneous information from the hand and fingers (Gibson, 1962).

It is known that skin hydration improves tactile discrimination ability, and this has been demonstrated during both static (Lévêque et al., 2000) and dynamic touch (Skedung et al., 2018). We also knew that the active ingredient included here in the foam increases skin hydration and induces vasodilatation, as compared to the same cosmetic foam without this ingredient (Anjac, personal communication). We demonstrate that the new psychophysical method we tested here found differences between the active and placebo groups, in terms of tactile acuity, and thus provides a high level of discrimination.

Recently, a similar method has been published where the short-term effects of applying skin moisturizing were assessed during dynamic touch, by analyzing subjects’ tactile perception ability using a test of texture discrimination (Skedung et al., 2018). In this study, participants had to judge a surface as being different from the reference one and/or to judge the reference to be the same when presented against itself, and authors quantified the percentage of correct responses. The fact that this study and ours was performed almost at the same time shows that there is a need nowadays to improve the old, classical tests to better evaluate tactile sensitivity, notably to assess the effects of treatments or remediation by training. The present method appears more sophisticated, since not only did the participant have to judge that the test texture was different from reference, but also to say in which way, i.e., with farther or closer grooves. Therefore, one should expect closer differences to be detected by the present method, which also allowed the extraction of statistical parameters that gave additional information, including the PSE and the uncertainty range. Therefore, the two methods constitute a real step towards a better estimation of tactile acuity and one may choose one or the other depending on the requirements and sensitivity of the experiment.

About the mechanisms responsible for the improvement in tactile discrimination, we can exclude an effect of protocol learning for two reasons: this should have impacted the participants from the placebo group, and more importantly, the participants were tested only two times, with a 1-month interval. Tactile learning of complex tasks requires a training based on multiple seances over a couple of weeks (Debowska et al., 2016), which was not the case here. The most reasonable hypothesis would have initially been that the improved performance in the active group was related to changes in skin mechanical properties, which we did not test here, but that has been largely documented, where moisturizing the stratum corneum has been shown to promote recovery of skin elasticity associated with improvement in tactile sensitivity (Lévêque et al., 2000; Bowden and McNulty, 2013; Skedung et al., 2018). However, in their recent study, Skedung et al. (2018) report a great variability in tactile sensitivity in elderly people, where high and low performers exhibited almost the same skin mechanical properties. In the present study, the two groups of subjects performed similarly before the month of cosmetic application and they were all exposed to the same amount of humectant or softening product, so that we can reasonably think that the mechanical properties of the skin were similar between the two groups of subjects after the month of cosmetic substance application. Interestingly, the same authors (Skedung et al., 2018) found that among the elderly participants, the higher performing group had a statistically higher density of Meissner corpuscles, which are cutaneous mechanoreceptors strongly involved in dynamic touch, such as in the present active task. All these considerations lead us to suggest that the active product used here may have promoted peripheral sensory regeneration after 1 month of application, as previously demonstrated to occur with various drugs such as hydrogen peroxide (Rieger and Sagasti, 2011) or neuropoietic cytokines (Feld et al., 2016), but this remains speculative and further studies are required to establish the properties of the present active product. Changes in tactile discrimination after 1 month of cosmetic substance application have been documented in elderly people using a new psychophysical method, to evaluate alterations in the range of a few micrometers. We suggest that this high level of discrimination validates our new test as being particularly useful for studying the effects of tactile rehabilitation and/or skin treatments in the elderly.

## Data Availability

The raw data supporting the conclusions of this manuscript will be made available by the authors, without undue reservation, to any qualified researcher.

## Ethics Statement

All participants gave their written, informed consent and the investigation was carried out in accordance with the Declaration of Helsinki.

## Author Contributions

This study was designed by ER-C, J-MA, and MC. Data collection and analysis were conducted by CD. PMMA sheets were designed and provided by P-HC and BW. The content of the manuscript was prepared by J-MA and ER-C.

## Conflict of Interest Statement

The authors declare that the research was conducted in the absence of any commercial or financial relationships that could be construed as a potential conflict of interest.
